# *Notes from the Field:* Increase in Hepatitis A Virus Infections — Marshall Islands, 2016–2017

**DOI:** 10.15585/mmwr.mm6717a5

**Published:** 2018-05-04

**Authors:** Megan G. Hofmeister, Jill A. McCready, Ruth Link-Gelles, Blanche Greene Cramer, Leisha D. Nolen, Helentina Garstang, DCHMS, Monique A. Foster

**Affiliations:** ^1^Division of Viral Hepatitis, National Center for HIV/AIDS, Viral Hepatitis, STD, and TB Prevention, CDC; ^2^Epidemic Intelligence Service, CDC; ^3^Pacific Island Health Officers’ Association; ^4^Division of Global Health Protection, Center for Global Health, CDC; ^5^Division of Preparedness and Emerging Infections, National Center for Emerging and Zoonotic Infectious Diseases, CDC; ^6^Department of Public Health, Ministry of Health and Human Services, Marshall Islands.

In mid-September 2016, a case of hepatitis A virus (HAV) infection was reported to the Marshall Islands Ministry of Health and Human Services (MOHHS). On November 4, MOHHS received laboratory confirmation of four additional cases, prompting activation of an outbreak investigation by the MOHHS Exposure Prevention Information Network (EPINet) team and solicitation of technical assistance from the Pacific Island Health Officers’ Association, the World Health Organization, and CDC. CDC began participating in the investigation by providing technical assistance remotely at that time. CDC provided remote assistance throughout the course of the investigation. In April 2017, the CDC-affiliated coauthors traveled to the Marshall Islands to provide in-person technical assistance.

To characterize the outbreak, the MOHHS EPINet Team, with assistance from CDC, conducted an investigation through in-person interviews and medical chart abstractions. A probable HAV outbreak case was defined as an acute illness with onset of any signs or symptoms consistent with acute viral hepatitis (e.g., fever, anorexia, nausea, vomiting, diarrhea, fatigue, dark urine, clay-colored stool, or abdominal pain) on or after September 1, 2016, and either jaundice or elevated serum aminotransferase levels; a confirmed case met the probable case definition and also had either a positive immunoglobulin M (IgM) antibody to HAV on laboratory testing or an epidemiologic link to a confirmed case.*

From September 2016 (epidemiologic week 37) through July 2017 (epidemiologic week 28), 194 outbreak-associated hepatitis A cases (168 confirmed and 26 probable) were reported by MOHHS (Figure). Illness onset dates ranged from September 12, 2016, through July 11, 2017. The median age of infected persons was 8 years (range = 2–76 years), 57% of patients were male, 91% were Marshallese, and 11% were hospitalized. No deaths were reported. Persons aged <25 years accounted for 90% of cases, and 92% of patients were residents of the capital, Majuro. The most commonly reported signs and symptoms were jaundice (92%), nausea (76%), anorexia (75%), and dark urine (68%). Clay-colored stool (10%) was less commonly reported.

**FIGURE:**
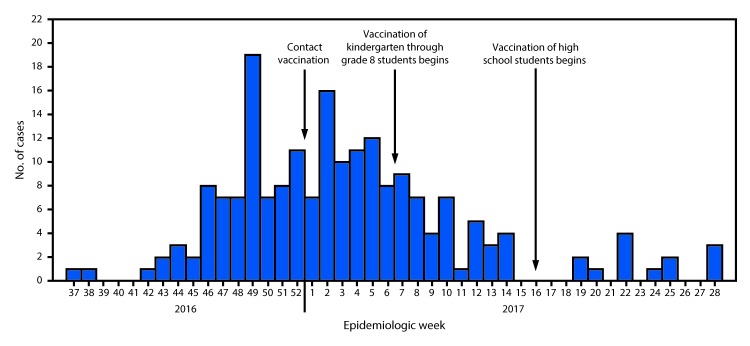
Number of confirmed and probable hepatitis A cases (N = 194) — Marshall Islands, September 2016–July 2017

Complete contact information was available for 102 (53%) patients. A total of 1,143 contacts were identified, with a mean of 11 contacts identified per patient (range = 2–60). Among the identified contacts, 902 (79%) received postexposure prophylaxis (PEP) with hepatitis A vaccine. Some contacts were identified outside the recommended PEP window of 14 days after exposure, and 14 contacts were infants who were too young to be vaccinated (*1*). Seven contacts refused vaccination.

The EPINet team disseminated public information about the outbreak and recommendations on hygiene and vaccination through radio shows, mass text messages, posters, and school presentations; developed standardized case reporting and interview tools; and expanded case finding through investigation of contacts. Hepatitis A vaccine is not currently included in the Marshall Islands routine childhood immunization schedule. Marshall Islands began immunization of contacts of patients with hepatitis A in January 2017 and then launched a comprehensive immunization campaign targeting school-aged children on Majuro in February 2017, which ultimately covered approximately 70% of the total kindergarten through eighth grade student population. Once the vaccine supply was replenished in April 2017, a second immunization campaign was directed at high school students aged 14–19 years on Majuro. In total, approximately 12,500 doses of hepatitis A vaccine were administered to school-aged children and adult contacts of patients in response to the outbreak. No additional cases were reported as of August 30, 2017.

Before this outbreak, the last HAV outbreak in the Marshall Islands occurred approximately 25 years ago. Since then, approximately five hepatitis A cases per year have been reported (MOHHS, unpublished data, 2017). HAV infection is typically acquired through fecal-oral transmission, either from direct person-to-person contact or consumption of contaminated food or water. In this outbreak, transmission occurred primarily through direct person-to-person contact, and despite extensive measures, the initial source of HAV infection was not identified.

HAV infection occurs in three distinct epidemiologic patterns (high, intermediate, and low endemicity) associated with hygiene and sanitation, access to clean drinking water, household crowding, and socioeconomic conditions (*2*). As socioeconomic conditions and sanitation improve, areas transition from high to intermediate endemicity, which is associated with an increased incidence of symptomatic clinical disease and potential for outbreaks. Hepatitis A–related hospitalizations and mortality also increase as the age of infection shifts from early childhood, when disease is typically asymptomatic or mild, to adolescence and adulthood, when illness is more likely to be severe (*2*).

Before this outbreak, HAV was thought to be endemic in the Marshall Islands; however, this outbreak demonstrates that the country might be undergoing an epidemiologic transition toward intermediate endemicity (*3*). Health officials are evaluating the potential costs and benefits of incorporating routine hepatitis A vaccination in Marshall Islands as a means of reducing ongoing transmission and preventing outbreaks.
